# *N*,*N*′-Dibenzyl-*N*′′-(4-bromo­benzo­yl)-*N*,*N*′-dimethyl­phospho­ric triamide

**DOI:** 10.1107/S1600536808004777

**Published:** 2008-02-27

**Authors:** Saeed Dehghanpour, Richard Welter, Aliou Hamady Barry, Farzaneh Tabasi

**Affiliations:** a Department of Chemistry, Alzahra University, Vanak, Tehran, Iran; b Laboratoire DECMET, UMR CNRS 7513, Université Louis Pasteur, Strasbourg, France; c Département de Chimie, Faculté des Sciences et Techniques, Université de Nouakchott, Nouakchott, Mauritania

## Abstract

In the title compound, C_23_H_25_BrN_3_O_2_P, the P atom has a distorted tetra­hedral coordination. In the crystal structure, the mol­ecules form centrosymmetric dimers *via* pairs of essentially linear N—H⋯O hydrogen bonds.

## Related literature

For the use of carbacyl­amido­phosphate, see: Barak *et al.* (2000 ▶); Burla *et al.* (1989 ▶); Gubina *et al.* (2000 ▶); Mallender *et al.* (2000 ▶); Trush *et al.* (2003 ▶). For related structures, see: Trush *et al.* (1999 ▶).
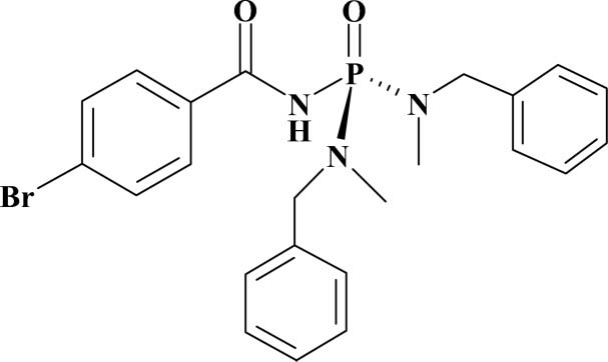


## Experimental

### 

#### Crystal data

C_23_H_25_BrN_3_O_2_P
*M* *_r_* = 486.34
Monoclinic, 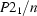

*a* = 9.0140 (4) Å
*b* = 13.2690 (5) Å
*c* = 19.377 (1) Å
β = 94.1500 (14)°
*V* = 2311.54 (18) Å^3^
*Z* = 4
Mo *K*α radiation
μ = 1.87 mm^−1^
*T* = 293 (2) K
0.11 × 0.09 × 0.08 mm

#### Data collection

Nonius KappaCCD diffractometer
Absorption correction: none
12951 measured reflections
5214 independent reflections
2593 reflections with *I* > 2σ(*I*)
*R*_int_ = 0.051

#### Refinement

*R*[*F* ^2^ > 2σ(*F* ^2^)] = 0.053
*wR*(*F* ^2^) = 0.135
*S* = 1.00
5214 reflections
271 parameters
H-atom parameters constrained
Δρ_max_ = 0.27 e Å^−3^
Δρ_min_ = −0.62 e Å^−3^

### 

Data collection: *COLLECT* (Nonius, 1998 ▶); cell refinement: *DENZO* (Otwinowski & Minor, 1997 ▶); data reduction: *DENZO*; program(s) used to solve structure: *SHELXS97* (Sheldrick, 2008 ▶); program(s) used to refine structure: *SHELXL97* (Sheldrick, 2008 ▶); molecular graphics: *PLATON* (Spek, 2003 ▶); software used to prepare material for publication: *SHELXL97*.

## Supplementary Material

Crystal structure: contains datablocks I, global. DOI: 10.1107/S1600536808004777/zl2095sup1.cif

Structure factors: contains datablocks I. DOI: 10.1107/S1600536808004777/zl2095Isup2.hkl

Additional supplementary materials:  crystallographic information; 3D view; checkCIF report

## Figures and Tables

**Table 1:** Hydrogen-bond geometry (Å, °)

*D*—H⋯*A*	*D*—H	H⋯*A*	*D*⋯*A*	*D*—H⋯*A*
N1—H1⋯O2^i^	0.86	1.99	2.845 (3)	170

Symmetry code: (i) 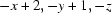.

**Fig. 1.**
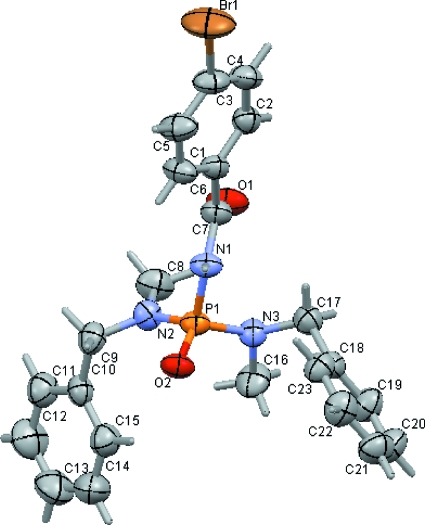
Molecular structure of I showing the atom-labelling scheme with thermal ellipsoids drawn at the 50% probability level.

**Fig. 2.**
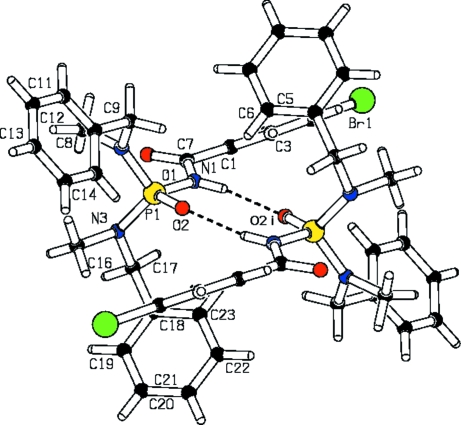
Representation of the hydrogen bonds in the structure of (I). For clarity, only the O2 atom in the second molecule is labeled (O2i), Symmetry code i: 2 - x, 1 - y, -z.

## supplementary crystallographic information

### Comment

Carbacylamidophosphate compounds have attracted substantial interest for many years. These compounds have been employed in coordination chemistry as chelating reagents for various metal ions *via* their =P(O)N(H)C(O)- moiety (Trush *et al.*, 2003; Gubina *et al.*, 2000), as prodrugs in pharmacology and as pesticides in agriculture (Barak *et al.*, 2000; Mallender *et al.*, 2000). A thorough knowledge of the structural properties of these compounds should be beneficial for a detailed understanding of their pharmacological effects. The title compound, (I), was prepared by the reaction of (pBr-C_6_H_5_)C(O)NHP(O)(Cl)_2_ with two molecules of methylmenzyl amine.

The crystal structure of (I) reveals that, in the molecular core unit C(O)NHP(O), the C(O) and P(O) oxygen atoms are in anti-positions to each other. The phosphorus centre has a slightly distorted tetrahedral coordination, mainly due to the presence of the different substituents. The N3–P1–N1 angle (105.20 (12)°) is narrower than the ideal tetrahedral angle of 109.5, whereas the opposite O2–P1–N3 angle (119.05 (13)°) is wider than the ideal tetrahedral angle. The P1–O2 bond length (1.474 (2) Å) is in good agreement with P–O distances in other carbacylamidophosphates (Trush *et al.*, 1999).

Examination of intermolecular distances indicates that the crystal structure of compound (I) consists of C_9_H_13_N_2_O_2_P units linked together *via* N—H···O hydrogen bonds into centrosymmetric dimers featuring eight-membered (OPNH)_2_ rings (Fig.2, Table 1).

### Experimental

Compound (I) was synthesized *via* the reaction of BrC_6_H_4_C(O)NHP(O)Cl_2_ with two molecules of methylbenzylamine in a 1:4 molar ratio. Methylbenzylamine was added dropwise to a mixture of BrC_6_H_4_C(O)NHP(O)Cl_2_ in chloroform while stirring at room temperature for 4 h. The product was filtered off and then washed with cold water. The compound was recrystallized from ethanol (yield 88%). Analysis, calculated for C_23_ H_25_ Br N_3_ O_2_ P: C 56.80, H 5.18, N 8.64%; found: C 56.81, H 5.19, N 8.64%.

### Refinement

H atoms were placed in idealized positions with C—H distances at 0.97, 0.96 and 0.93 Å for CH_2_, CH_3_ and aromatic CH groups, respectively using a riding model. *U*_iso_(H) for H was assigned as 1.2 *U*_eq_(Ci) of the attached C atoms (1.5 for methyl). No absorption correction was applied due to the small cystal size and the sufficiently low µ value.

### Crystal data

**Table:**

C_23_H_25_BrN_3_O_2_P	*F*_000_ = 1000
*M**_r_* = 486.34	*D*_x_ = 1.397 Mg m^−^^3^
Monoclinic, *P*2_1_/*n*	Mo *K*α radiation λ = 0.71073 Å
Hall symbol: -P 2yn	Cell parameters from 10293 reflections
*a* = 9.0140 (4) Å	θ = 1.0–27.5º
*b* = 13.2690 (5) Å	µ = 1.87 mm^−^^1^
*c* = 19.3770 (10) Å	*T* = 293 (2) K
β = 94.1500 (14)º	Prism, colorless
*V* = 2311.54 (18) Å^3^	0.11 × 0.09 × 0.08 mm
*Z* = 4	

### Data collection

**Table:**

Nonius KappaCCD diffractometer	2593 reflections with *I* > 2σ(*I*)
Radiation source: fine-focus sealed tube	*R*_int_ = 0.051
Monochromator: graphite	θ_max_ = 27.5º
*T* = 293(2) K	θ_min_ = 1.9º
π scans	*h* = −11→10
Absorption correction: none	*k* = −17→14
12951 measured reflections	*l* = −17→25
5214 independent reflections	

### Refinement

**Table:**

Refinement on *F*^2^	Secondary atom site location: difference Fourier map
Least-squares matrix: full	Hydrogen site location: inferred from neighbouring sites
*R*[*F*^2^ > 2σ(*F*^2^)] = 0.053	H-atom parameters constrained
*wR*(*F*^2^) = 0.135	*w* = 1/[σ^2^(*F*_o_^2^) + (0.052*P*)^2^ + 0.3719*P*] where *P* = (*F*_o_^2^ + 2*F*_c_^2^)/3
*S* = 1.00	(Δ/σ)_max_ = 0.001
5214 reflections	Δρ_max_ = 0.27 e Å^−^^3^
271 parameters	Δρ_min_ = −0.62 e Å^−^^3^
Primary atom site location: structure-invariant direct methods	Extinction correction: none

### Special details

**Table:**

Geometry. All e.s.d.'s (except the e.s.d. in the dihedral angle between two l.s. planes) are estimated using the full covariance matrix. The cell e.s.d.'s are taken into account individually in the estimation of e.s.d.'s in distances, angles and torsion angles; correlations between e.s.d.'s in cell parameters are only used when they are defined by crystal symmetry. An approximate (isotropic) treatment of cell e.s.d.'s is used for estimating e.s.d.'s involving l.s. planes.
Refinement. Refinement of *F*^2^ against ALL reflections. The weighted *R*-factor *wR* and goodness of fit *S* are based on *F*^2^, conventional *R*-factors *R* are based on *F*, with *F* set to zero for negative *F*^2^. The threshold expression of *F*^2^ > σ(*F*^2^) is used only for calculating *R*-factors(gt) *etc*. and is not relevant to the choice of reflections for refinement. *R*-factors based on *F*^2^ are statistically about twice as large as those based on *F*, and *R*- factors based on ALL data will be even larger.

### Fractional atomic coordinates and isotropic or equivalent isotropic displacement parameters (Å^2^)

**Table:**

	*x*	*y*	*z*	*U*_iso_*/*U*_eq_	
Br1	0.97528 (7)	0.99976 (3)	0.19720 (3)	0.1095 (3)	
P1	0.97988 (9)	0.37741 (5)	0.08475 (4)	0.0427 (2)	
O1	0.8889 (3)	0.48944 (15)	0.21515 (13)	0.0676 (7)	
O2	1.0335 (2)	0.37714 (14)	0.01466 (10)	0.0493 (5)	
N1	0.9570 (3)	0.49921 (14)	0.10487 (13)	0.0453 (6)	
H1	0.9661	0.5414	0.0717	0.054*	
N2	1.0988 (3)	0.32013 (17)	0.13878 (12)	0.0478 (7)	
N3	0.8203 (3)	0.32336 (16)	0.09738 (13)	0.0474 (6)	
C1	0.9380 (3)	0.6529 (2)	0.17215 (15)	0.0440 (7)	
C2	0.8517 (3)	0.7031 (2)	0.21718 (16)	0.0505 (8)	
H2	0.7872	0.6665	0.2429	0.061*	
C3	0.8593 (4)	0.8061 (2)	0.22469 (17)	0.0588 (9)	
H3	0.7986	0.8395	0.2541	0.071*	
C4	0.9584 (4)	0.8585 (2)	0.18784 (18)	0.0615 (10)	
C5	1.0478 (4)	0.8100 (2)	0.14387 (19)	0.0682 (10)	
H5	1.1152	0.8465	0.1196	0.082*	
C6	1.0366 (4)	0.7066 (2)	0.13596 (17)	0.0564 (9)	
H6	1.0961	0.6733	0.1060	0.068*	
C7	0.9257 (3)	0.5404 (2)	0.16691 (17)	0.0486 (8)	
C8	1.0706 (4)	0.2882 (3)	0.20873 (17)	0.0669 (10)	
H8A	0.9697	0.3039	0.2176	0.100*	
H8B	1.1373	0.3228	0.2416	0.100*	
H8C	1.0861	0.2168	0.2131	0.100*	
C9	1.2491 (4)	0.2979 (2)	0.11888 (18)	0.0566 (9)	
H9A	1.3200	0.3171	0.1566	0.068*	
H9B	1.2686	0.3390	0.0792	0.068*	
C10	1.2746 (3)	0.1886 (2)	0.10116 (17)	0.0506 (8)	
C11	1.3680 (4)	0.1286 (3)	0.14281 (19)	0.0680 (10)	
H11	1.4111	0.1543	0.1842	0.082*	
C12	1.3988 (5)	0.0301 (3)	0.1237 (3)	0.0867 (13)	
H12	1.4631	−0.0095	0.1520	0.104*	
C13	1.3348 (5)	−0.0078 (3)	0.0640 (3)	0.0903 (14)	
H13	1.3546	−0.0738	0.0513	0.108*	
C14	1.2410 (5)	0.0505 (3)	0.0221 (2)	0.0830 (12)	
H14	1.1980	0.0241	−0.0191	0.100*	
C15	1.2100 (4)	0.1478 (3)	0.04062 (19)	0.0646 (10)	
H15	1.1450	0.1865	0.0122	0.078*	
C16	0.8132 (4)	0.2134 (2)	0.0867 (2)	0.0710 (11)	
H16A	0.9095	0.1846	0.0981	0.107*	
H16B	0.7829	0.1994	0.0391	0.107*	
H16C	0.7425	0.1848	0.1158	0.107*	
C17	0.6770 (3)	0.3738 (2)	0.08216 (17)	0.0545 (8)	
H17A	0.6872	0.4439	0.0959	0.065*	
H17B	0.6043	0.3433	0.1102	0.065*	
C18	0.6182 (3)	0.3696 (2)	0.00736 (16)	0.0463 (8)	
C19	0.5215 (4)	0.2942 (3)	−0.01586 (19)	0.0655 (10)	
H19	0.4908	0.2463	0.0152	0.079*	
C20	0.4698 (4)	0.2890 (3)	−0.0847 (2)	0.0781 (11)	
H20	0.4056	0.2374	−0.0998	0.094*	
C21	0.5125 (4)	0.3591 (3)	−0.13033 (19)	0.0726 (11)	
H21	0.4761	0.3562	−0.1764	0.087*	
C22	0.6101 (4)	0.4348 (3)	−0.10826 (19)	0.0665 (10)	
H22	0.6410	0.4823	−0.1395	0.080*	
C23	0.6611 (4)	0.4394 (2)	−0.03991 (19)	0.0574 (9)	
H23	0.7261	0.4907	−0.0251	0.069*	

### Atomic displacement parameters (Å^2^)

**Table:**

	*U*^11^	*U*^22^	*U*^33^	*U*^12^	*U*^13^	*U*^23^
Br1	0.1798 (6)	0.0408 (2)	0.1100 (5)	−0.0035 (2)	0.0253 (4)	−0.0180 (2)
P1	0.0553 (5)	0.0330 (4)	0.0398 (5)	0.0004 (4)	0.0042 (4)	0.0012 (3)
O1	0.108 (2)	0.0516 (14)	0.0455 (14)	−0.0064 (12)	0.0206 (14)	0.0020 (11)
O2	0.0716 (14)	0.0369 (11)	0.0401 (13)	0.0037 (9)	0.0092 (11)	0.0006 (9)
N1	0.0654 (17)	0.0327 (13)	0.0383 (15)	−0.0008 (11)	0.0068 (13)	0.0019 (10)
N2	0.0522 (17)	0.0476 (14)	0.0432 (16)	0.0058 (12)	0.0009 (13)	0.0108 (12)
N3	0.0476 (16)	0.0404 (14)	0.0542 (17)	−0.0004 (11)	0.0035 (13)	0.0044 (12)
C1	0.0524 (19)	0.0436 (17)	0.0356 (18)	0.0029 (14)	−0.0001 (15)	−0.0034 (14)
C2	0.052 (2)	0.0533 (19)	0.0466 (19)	−0.0017 (15)	0.0035 (16)	−0.0025 (16)
C3	0.071 (3)	0.053 (2)	0.051 (2)	0.0103 (17)	0.0030 (18)	−0.0144 (17)
C4	0.087 (3)	0.0387 (18)	0.058 (2)	0.0037 (17)	0.001 (2)	−0.0077 (16)
C5	0.091 (3)	0.053 (2)	0.063 (2)	−0.0175 (18)	0.016 (2)	−0.0092 (18)
C6	0.073 (2)	0.0428 (18)	0.056 (2)	−0.0014 (16)	0.0195 (18)	−0.0081 (16)
C7	0.060 (2)	0.0449 (17)	0.042 (2)	0.0006 (15)	0.0078 (16)	−0.0022 (16)
C8	0.090 (3)	0.063 (2)	0.047 (2)	0.0128 (19)	0.0006 (19)	0.0097 (17)
C9	0.051 (2)	0.0508 (19)	0.067 (2)	−0.0036 (15)	−0.0023 (17)	0.0058 (17)
C10	0.0425 (19)	0.0478 (18)	0.062 (2)	−0.0007 (14)	0.0061 (17)	0.0063 (17)
C11	0.064 (2)	0.063 (2)	0.075 (3)	0.0067 (18)	−0.006 (2)	0.005 (2)
C12	0.092 (3)	0.068 (3)	0.099 (4)	0.028 (2)	−0.006 (3)	0.016 (3)
C13	0.096 (3)	0.063 (3)	0.113 (4)	0.017 (2)	0.012 (3)	−0.011 (2)
C14	0.083 (3)	0.078 (3)	0.087 (3)	0.005 (2)	0.004 (2)	−0.021 (2)
C15	0.062 (2)	0.062 (2)	0.068 (3)	0.0057 (18)	−0.004 (2)	0.0013 (19)
C16	0.074 (3)	0.0398 (18)	0.098 (3)	−0.0097 (16)	−0.003 (2)	0.0053 (19)
C17	0.052 (2)	0.058 (2)	0.054 (2)	0.0010 (16)	0.0072 (17)	−0.0010 (16)
C18	0.0409 (18)	0.0485 (18)	0.050 (2)	0.0054 (14)	0.0055 (15)	−0.0038 (15)
C19	0.061 (2)	0.070 (2)	0.064 (3)	−0.0133 (18)	0.0004 (19)	0.005 (2)
C20	0.081 (3)	0.073 (2)	0.077 (3)	−0.020 (2)	−0.015 (2)	0.001 (2)
C21	0.083 (3)	0.077 (3)	0.055 (2)	0.007 (2)	−0.011 (2)	−0.004 (2)
C22	0.071 (3)	0.068 (2)	0.059 (3)	0.0012 (19)	−0.002 (2)	0.0139 (19)
C23	0.059 (2)	0.050 (2)	0.062 (2)	−0.0029 (15)	−0.0046 (18)	0.0030 (17)

### Geometric parameters (Å, °)

**Table:**

Br1—C4	1.889 (3)	C10—C11	1.377 (4)
P1—O2	1.474 (2)	C10—C15	1.382 (4)
P1—N2	1.631 (2)	C11—C12	1.392 (5)
P1—N3	1.642 (2)	C11—H11	0.9300
P1—N1	1.679 (2)	C12—C13	1.353 (6)
O1—C7	1.218 (4)	C12—H12	0.9300
N1—C7	1.368 (4)	C13—C14	1.369 (6)
N1—H1	0.8600	C13—H13	0.9300
N2—C8	1.460 (4)	C14—C15	1.375 (5)
N2—C9	1.465 (4)	C14—H14	0.9300
N3—C17	1.465 (4)	C15—H15	0.9300
N3—C16	1.474 (4)	C16—H16A	0.9600
C1—C6	1.370 (4)	C16—H16B	0.9600
C1—C2	1.381 (4)	C16—H16C	0.9600
C1—C7	1.501 (4)	C17—C18	1.508 (4)
C2—C3	1.376 (4)	C17—H17A	0.9700
C2—H2	0.9300	C17—H17B	0.9700
C3—C4	1.372 (5)	C18—C23	1.377 (4)
C3—H3	0.9300	C18—C19	1.381 (4)
C4—C5	1.374 (5)	C19—C20	1.382 (5)
C5—C6	1.385 (4)	C19—H19	0.9300
C5—H5	0.9300	C20—C21	1.359 (5)
C6—H6	0.9300	C20—H20	0.9300
C8—H8A	0.9600	C21—C22	1.382 (5)
C8—H8B	0.9600	C21—H21	0.9300
C8—H8C	0.9600	C22—C23	1.371 (5)
C9—C10	1.512 (4)	C22—H22	0.9300
C9—H9A	0.9700	C23—H23	0.9300
C9—H9B	0.9700		
			
O2—P1—N2	110.25 (13)	C11—C10—C15	118.3 (3)
O2—P1—N3	119.05 (13)	C11—C10—C9	121.2 (3)
N2—P1—N3	104.06 (12)	C15—C10—C9	120.4 (3)
O2—P1—N1	105.69 (12)	C10—C11—C12	120.8 (3)
N2—P1—N1	112.69 (13)	C10—C11—H11	119.6
N3—P1—N1	105.20 (12)	C12—C11—H11	119.6
C7—N1—P1	128.7 (2)	C13—C12—C11	119.7 (4)
C7—N1—H1	115.7	C13—C12—H12	120.1
P1—N1—H1	115.7	C11—C12—H12	120.1
C8—N2—C9	114.4 (2)	C12—C13—C14	120.3 (4)
C8—N2—P1	125.5 (2)	C12—C13—H13	119.9
C9—N2—P1	120.1 (2)	C14—C13—H13	119.9
C17—N3—C16	113.3 (2)	C13—C14—C15	120.3 (4)
C17—N3—P1	122.65 (19)	C13—C14—H14	119.8
C16—N3—P1	116.2 (2)	C15—C14—H14	119.8
C6—C1—C2	119.3 (3)	C14—C15—C10	120.6 (3)
C6—C1—C7	122.0 (3)	C14—C15—H15	119.7
C2—C1—C7	118.7 (3)	C10—C15—H15	119.7
C3—C2—C1	121.3 (3)	N3—C16—H16A	109.5
C3—C2—H2	119.3	N3—C16—H16B	109.5
C1—C2—H2	119.3	H16A—C16—H16B	109.5
C4—C3—C2	118.5 (3)	N3—C16—H16C	109.5
C4—C3—H3	120.7	H16A—C16—H16C	109.5
C2—C3—H3	120.7	H16B—C16—H16C	109.5
C3—C4—C5	121.3 (3)	N3—C17—C18	114.8 (3)
C3—C4—Br1	120.2 (3)	N3—C17—H17A	108.6
C5—C4—Br1	118.5 (3)	C18—C17—H17A	108.6
C4—C5—C6	119.4 (3)	N3—C17—H17B	108.6
C4—C5—H5	120.3	C18—C17—H17B	108.6
C6—C5—H5	120.3	H17A—C17—H17B	107.5
C1—C6—C5	120.2 (3)	C23—C18—C19	118.1 (3)
C1—C6—H6	119.9	C23—C18—C17	121.2 (3)
C5—C6—H6	119.9	C19—C18—C17	120.7 (3)
O1—C7—N1	122.5 (3)	C18—C19—C20	120.7 (3)
O1—C7—C1	121.5 (3)	C18—C19—H19	119.6
N1—C7—C1	116.0 (3)	C20—C19—H19	119.6
N2—C8—H8A	109.5	C21—C20—C19	120.2 (3)
N2—C8—H8B	109.5	C21—C20—H20	119.9
H8A—C8—H8B	109.5	C19—C20—H20	119.9
N2—C8—H8C	109.5	C20—C21—C22	120.0 (3)
H8A—C8—H8C	109.5	C20—C21—H21	120.0
H8B—C8—H8C	109.5	C22—C21—H21	120.0
N2—C9—C10	114.3 (2)	C23—C22—C21	119.5 (3)
N2—C9—H9A	108.7	C23—C22—H22	120.3
C10—C9—H9A	108.7	C21—C22—H22	120.3
N2—C9—H9B	108.7	C22—C23—C18	121.5 (3)
C10—C9—H9B	108.7	C22—C23—H23	119.3
H9A—C9—H9B	107.6	C18—C23—H23	119.3
			
O2—P1—N1—C7	−171.6 (3)	C2—C1—C7—O1	−27.1 (4)
N2—P1—N1—C7	−51.2 (3)	C6—C1—C7—N1	−30.6 (4)
N3—P1—N1—C7	61.6 (3)	C2—C1—C7—N1	152.3 (3)
O2—P1—N2—C8	−167.1 (2)	C8—N2—C9—C10	76.2 (3)
N3—P1—N2—C8	−38.3 (3)	P1—N2—C9—C10	−104.6 (3)
N1—P1—N2—C8	75.1 (3)	N2—C9—C10—C11	−111.6 (3)
O2—P1—N2—C9	13.8 (2)	N2—C9—C10—C15	71.6 (4)
N3—P1—N2—C9	142.6 (2)	C15—C10—C11—C12	1.2 (5)
N1—P1—N2—C9	−104.0 (2)	C9—C10—C11—C12	−175.6 (3)
O2—P1—N3—C17	−81.9 (3)	C10—C11—C12—C13	−0.8 (6)
N2—P1—N3—C17	154.9 (2)	C11—C12—C13—C14	0.5 (7)
N1—P1—N3—C17	36.2 (3)	C12—C13—C14—C15	−0.5 (7)
O2—P1—N3—C16	64.9 (3)	C13—C14—C15—C10	1.0 (6)
N2—P1—N3—C16	−58.3 (3)	C11—C10—C15—C14	−1.3 (5)
N1—P1—N3—C16	−177.0 (2)	C9—C10—C15—C14	175.6 (3)
C6—C1—C2—C3	2.2 (5)	C16—N3—C17—C18	−65.4 (3)
C7—C1—C2—C3	179.4 (3)	P1—N3—C17—C18	82.3 (3)
C1—C2—C3—C4	−2.0 (5)	N3—C17—C18—C23	−85.4 (4)
C2—C3—C4—C5	0.5 (5)	N3—C17—C18—C19	93.4 (3)
C2—C3—C4—Br1	−179.1 (2)	C23—C18—C19—C20	0.2 (5)
C3—C4—C5—C6	0.7 (6)	C17—C18—C19—C20	−178.7 (3)
Br1—C4—C5—C6	−179.6 (3)	C18—C19—C20—C21	−0.7 (6)
C2—C1—C6—C5	−0.9 (5)	C19—C20—C21—C22	1.2 (6)
C7—C1—C6—C5	−178.0 (3)	C20—C21—C22—C23	−1.1 (6)
C4—C5—C6—C1	−0.5 (5)	C21—C22—C23—C18	0.5 (5)
P1—N1—C7—O1	−10.8 (5)	C19—C18—C23—C22	−0.1 (5)
P1—N1—C7—C1	169.7 (2)	C17—C18—C23—C22	178.8 (3)
C6—C1—C7—O1	150.0 (3)		

### Hydrogen-bond geometry (Å, °)

**Table:**

*D*—H···*A*	*D*—H	H···*A*	*D*···*A*	*D*—H···*A*
N1—H1···O2^i^	0.86	1.99	2.845 (3)	170.4

Symmetry codes: (i) −*x*+2, −*y*+1, −*z*.
